# Implementation of perinatal collaborative care: a health services approach to perinatal depression care

**DOI:** 10.1017/S1463423620000110

**Published:** 2020-09-10

**Authors:** Emily S. Miller, Rebekah Jensen, M. Camille Hoffman, Lauren M. Osborne, Katherine McEvoy, Nancy Grote, Eydie L. Moses-Kolko

**Affiliations:** 1 Department of Obstetrics and Gynecology, School of Medicine, Northwestern University Feinberg, Chicago, IL, USA; 2 Department of Obstetrics and Gynecology, School of Medicine, University of Colorado, Boulder, CO, USA; 3 Department of Psychiatry and Behavioral Sciences, Women’s Mood Disorders Center, School of Medicine, Johns Hopkins University, Baltimore, MD, USA; 4 Department of Obstetrics and Gynecology, Johns Hopkins University, Baltimore, MD, USA; 5 School of Social Work, University of Washington, Seattle, WA, USA; 6 Department of Psychiatry, Western Psychiatric Institute and Clinic, University of Pittsburgh, Pittsburgh, PA, USA

**Keywords:** collaborative care, integrated care, perinatal mental health, perinatal psychiatry

## Abstract

**Aim:**

Our objective was to integrate lessons learned from perinatal collaborative care programs across the United States, recognizing the diversity of practice settings and patient populations, to provide guidance on successful implementation.

**Background:**

Collaborative care is a health services delivery system that integrates behavioral health care into primary care. While efficacious, effectiveness requires rigorous attention to implementation to ensure adherence to the core evidence base.

**Methods:**

Implementation strategies are divided into three pragmatic stages: preparation, program launch, and program growth and sustainment; however, these steps are non-linear and dynamic.

**Findings:**

The discussion that follows is not meant to be prescriptive; rather, all implementation tasks should be thoughtfully tailored to the unique needs and setting of the obstetric community and patient population. In particular, we are aware that implementation on the level described here assumes commitment of both effort and money on the part of clinicians, administrators, and the health system, and that such financial resources are not always available. We conclude with synthesis of a survey of existing collaborative care programs to identify implementation practices of existing programs.

## Introduction

Collaborative care (CC) titrates the delivery of behavioral health care to the treatment intensity required for a given patient. CC typically begins with provision of psychoeducation about depression and initiation of evidence-based brief psychotherapy and/or pharmacotherapy. If the patient does not respond to the initial line of treatment, care is augmented (Katon, 2003). Very often the primary care physician can implement pharmacotherapy changes, thereby efficiently reserving specialty mental health care by a psychiatrist for those patients who do not respond to earlier steps in the treatment algorithm. Proposed benefits of CC include improved access to behavioral health, patient-centered care, receipt of behavioral and physical health care in the same familiar setting, and improved clinical outcomes. These benefits are achieved through adherence to five core principles: patient-centered team care, population-based care, measurement-based treatment to target, evidence-based care, and accountable care (Huffman *et al*., 2014). A care manager serves as the lynchpin of the CC program, facilitating longitudinal symptom monitoring and specialist-provided stepped care recommendations. Meta-analytic data consistently find evidence of improved depressive outcomes when comparing CC to usual care (Archer *et al*., 2012; Thota *et al*., 2012). Furthermore, myriad studies have demonstrated CC not only to be efficacious, but also effective when implemented in real-world settings (Grypma *et al*., 2006; Reiss-Brennan *et al*., 2010; Solberg *et al*., 2010; Zivin *et al*., 2010). Moreover, CC in a primary care setting is cost-effective (Katon *et al*., 2005; Unutzer *et al*., 2008; Katon *et al*., 2012).

Two randomized controlled trials have examined CC in the perinatal setting. Grote *et al.* (2015) randomized 168 pregnant women in the Seattle-King County Public Health System with probable major depressive disorder or dysthymia to receive either the MOMCare CC intervention or augmented usual care (Maternity Support Services, MSS-Plus). They found that MOMCare led to a reduction in depression severity as well as higher rates of adherence to care and depression remission. Furthermore, the MOMCare program was found to be cost-effective if the value of a depression-free day was estimated to be at least $20. Melville *et al.* (2014) similarly compared a CC model to usual care in obstetric/gynecological patients (7% of whom were pregnant) with major depression and/or dysthymia and identified improved functioning as well as an increase in depression remission in women randomized to the CC arm. Collectively, these data demonstrate perinatal CC to be efficacious. Importantly, these trials were conducted in two disparate care settings: MOMCare was conducted in a federally qualified health center, a system serving predominantly low-income women with a focus on primary care. Dr. Melville’s trial was conducted in two academic urban obstetrics and gynecology clinics serving a socioeconomically diverse population with a mix of insurers but under a more traditional obstetrics model. The demonstrated efficacy in each of these settings enhances the likelihood of external generalizability to a broad array of populations and practice types.

Despite these promising data, scant information is available regarding implementation of perinatal CC in the absence of the infrastructure and resources garnered in randomized trials (Lomonaco-Haycraft *et al*., 2018). This is particularly salient in the perinatal setting given the nuances in the obstetric health system, clinician comfort with the management of depression, and patient-level barriers (and facilitators) to engaging in mental health care. Moreover, how CC billing codes are conceptualized in bundled maternity payments remains an area of uncertainty with respect to the systems-level cost of implementation.

## Preparation

Laying the foundation for CC through effective preparation takes time and is paramount to a successful program. The AIMS Center at the University of Washington offers a comprehensive Implementation Guide focused on implementation of CC in the primary care setting (http://aims.uw.edu/collaborative-care/implementation-guide). Many of these resources can prove beneficial in planning for programmatic development in the perinatal setting.

### Needs and strengths assessment

The first step in program preparation is a data-driven needs assessment to determine the obstetric patient volume within the care system and the magnitude of the at-risk population. Specifically, depression screening patterns of obstetric providers must be understood. While multiple national professional organizations endorse both antenatal and postpartum depression screening (American College of Obstetrics and Gynecology, 2008; Siu *et al*., 2016; Kendig *et al*., 2017), organizations or individual practice groups may not have yet successfully implemented universal screening practices. Often after the woman is screened and meets criteria for depression, those who completed the screening do not know the most effective resources to help her. Behavioral health services are rarely co-located, and they are often fragmented throughout the community and have long wait-lists. Thus, while a CC program may improve screening rates, understanding the existing culture allows for better estimations of anticipated antenatal and postpartum mental health needs.

Obstetric care providers’ perspectives on their scope of practice are another critical component of the needs assessment. CC requires the obstetric clinician to be the primary prescriber of pharmacotherapy, with the psychiatrist serving primarily in a consultative role. The American College of Obstetricians and Gynecologists supports the role of obstetric care providers in provision of psychopharmacologic agents (American College of Obstetrics and Gynecology, 2015), but it is important to gauge both obstetricians’ willingness to engage in the CC model and the scope(s) of psychiatric morbidities the obstetricians are comfortable co-managing with the CC team. Similarly, understanding existing behavioral health resources serving the target patient population, with their strengths and limitations, is a critical component to tailoring the program to meet existing clinical gaps. Finally, it is critical to assess readiness to change, measured by obstetrician motivation to become involved in behavioral health issues, satisfaction with the existing model, and recognition that mental health is an important aspect of perinatal health.

### Behavioral care manager

The behavioral care manager is the lynchpin of CC, and the success of the program is tightly linked to this role. Care managers are behavioral health professionals, often a clinical social worker, psychologist, or nurse by training, and serve as the primary contact point for patients and obstetricians alike. The core responsibilities for the care manager are described in Table 1. Licensure to conduct evidence-based brief psychotherapy is helpful to facilitate care and achieve revenue generation when CC codes are not available for use (see *Funding Strategy* below).

**Table 1.** Core responsibilities of a behavioral care manager

Referrals	Accept referrals via various providers and communication methods
Enrollment	Lead the patient enrollment process, gathering and recording relevant information in the patient tracking tool
Assessment	Conduct brief clinical assessment to establish probable diagnosis, risk of harm, level of need, and preliminary treatment plan
Treatment	Consult with a collaborative care psychiatrist regarding pharmacotherapy; provide evidence-based brief psychotherapy; provide therapeutic support to patients as necessary
Monitoring	Ensure treatment to target: monitor self-reported screenings and follow protocols to reach therapeutic dose of medication and/or therapy frequency
Communication	Facilitate communication between obstetric providers and patients to ensure collaboration and functional treatment plan
Administration	Identify challenges in work flow to be addressed.

Knowledge of the CC model is essential, and several trainings on integrated and CC are available for the behavioral care manager (Table 2). While some background knowledge in women’s health is useful, a targeted orientation can be provided to fill in any knowledge voids. Relevant training topics may include a brief overview of standard prenatal care, introduction to perinatal complications and perinatal loss, unique features of perinatal mood disorders (including epidemiology, clinical presentation, and perinatal psychopharmacology), and, in some CC models, training in evidence-based brief psychotherapy (e.g., problem-solving therapy^13^ or brief interpersonal psychotherapy^12^). It is essential that the care manager understands the culture of the local obstetric clinic, including existing patient care and clinical work flow. The following skills are assets to the role of a CC manager: excellent communication, leadership, team orientation, flexibility, enthusiasm, initiative, teaching/training, attention to detail, generosity, and warmth.

**Table 2.** Integrated care training programs for care managers

Institution	Training	Duration of training	Modality
University of Washington AIMS Center	Care Manager Role – Clinical Skills	40 min	Online video
American Psychiatric Association	Foundational Concepts of the Care Manager	90 min	Online modules
University of Massachusetts Medical School	Primary Care Behavioral Health Certificate Program	22 e-learning modules, each takes 90–120 min	Self-guided, web-based
University of Michigan	Certificate in Integrated Behavioral Health and Primary Care	34 h (50 of continuing education credit)	Web-based – mix of live and pre-recorded lectures
Radford University (Virginia)	Behavioral Health and Integration Training Institute	40 h of continuing education in one week	In-person
Farleigh Dickinson University	Certificate Program in Integrated Primary Care	80 h of continuing education – 5 units with four modules each, completed over 20 weeks	Web-based

### Stakeholders and champions

A multidisciplinary approach is essential to CC implementation. In general, the planning team should include the CC manager and clinical and administrative leaders from both obstetrics and psychiatry. Patients can also be engaged in program development; focus groups to establish patient needs and patient-identified barriers to engaging in mental health treatment may guide specific programmatic foci. Clinical stakeholders should include a representative from each obstetric clinic served, to ensure that their specific needs are met. The perinatal psychiatrist and therapist who will be involved in clinical consultation and supervision should provide their perspectives on an anticipated work flow that serves the interest of the obstetric patient population involving both short-term treatment and team collaboration to optimize access to services for patients in greatest need.

From an administrative perspective, practice managers and, in an academic setting, department administrators, offer important perspectives ranging from physical space availability (see *Select Physical Space* below), insurance alignment, and clear delineation of responsibilities for pre-certification for mental health benefits, billing, and tracking the electronic medical record (EMR) charge capture. The newly developed CC codes (Centers for Medicare and Medicaid Services, 2019) offer financial support for implementation of the CC model and should be thoughtfully incorporated. Of note, maternity care services are, via some insurers, paid for utilizing bundled payments. How these CC codes integrate with systems of bundled payment remains unclear.

Divergent reimbursement practices represent a common barrier to implementation of CC (Kathol *et al*., 2010). Often insurances accepted by the obstetric clinics do not align with those accepted by mental health providers. Obstetricians may be less likely to participate in a CC program when only a subsection of their patients, those covered by select plans, can receive mental health care. Development of creative solutions to enable universal participation in CC for all obstetric patients optimizes the likelihood of success.

The CC ‘team’ should eventually grow through incorporation of practice champions among the front desk and clinical staff and modeling of ‘best practices’ by the team. Engagement of champions surpasses simple buy-in but rather provides the obstetric practice a sense of ownership in the CC model, which includes opportunities for regular feedback and frequent communication. Early team and champion meetings can clarify the model of CC, define the scope of the program, and highlight needs for additional education and training.

### Screening tools

Evidence-based screenings are another key feature of CC. At a minimum, the implementation team should select validated screening(s) for depression (e.g., Patient Health Questionnaire-9 (PHQ-9) (Kroenke *et al*., 2001), Edinburgh Postnatal Depression Scale (EPDS) (Cox *et al*., 1987), anxiety (e.g., Generalized Anxiety Disorder-7 item scale (GAD-7) (Spitzer *et al*., 2006), Perinatal Anxiety Screening Scale (PASS) (Somerville *et al*., 2014)), and bipolar disorder (e.g., Mood Disorder Questionnaire (MDQ) (Hirschfeld *et al*., 2000), WHIPLASHED clinician administered interview (Mahmoud *et al*., 2019)). It is recommended that all obstetric patients are screened by the obstetric clinicians both antenatally and postpartum using a validated screening tool (American College of Obstetrics and Gynecology, 2015; Siu *et al*., 2016). The same instrument utilized by the obstetrician for screening should be repeated at subsequent behavioral health visits to monitor for improvement. To support treatment to target (i.e., achieving remission of symptoms), particularly in the context of postpartum care when in-person visits are less frequent, symptom monitoring may include serial electronic delivery of the symptom screen(s) using Health Insurance Portability and Accountability Act (HIPAA)-compliant email surveys or phone calls with a frequency of screens informed by the severity of symptoms endorsed. Telephone-based visits to assess symptoms can also be used. This surveillance allows for discussion and adjustment of the care plan at intervals more frequent than in-person office visits.

The GAD-7, PASS, or high scores (2-3) on the EPDS questions 4, 5, and 6, can quantify anxiety symptoms, as depression and anxiety are highly comorbid in the perinatal period. For women with a positive screen for anxiety, following anxiety symptomatology via electronic screens, similar to the PHQ-9 described above, may identify exacerbations in symptoms that require intervention.

The MDQ or WHIPLASHED can help to rule out bipolar disorder, which is especially important if an obstetrician or midwife is considering starting an antidepressant. While a significant minority of perinatal women with a positive depression screen will have a diagnosis of bipolar disorder (Wisner *et al*., 2013), screening for bipolar disorder is not routinely performed by obstetricians. Systematic baseline screening enables identification of women for whom psychiatric consultation is prudent prior to initiation of pharmacotherapy.

Some programs screen enrolled patients with the Adverse Childhood Experience Questionnaire and the Post-traumatic stress disorder Checklist – Civilian Version. Correlation between trauma and perinatal depression is strong (Garabedian *et al*., 2011; Dennis and Vigod, 2013; Seng *et al*., 2013), and these two tools quickly provide helpful clinical information to inform risk and anticipated clinical outcome trajectories (Grote *et al*., 2016).

### Population served

**Figure 1.**
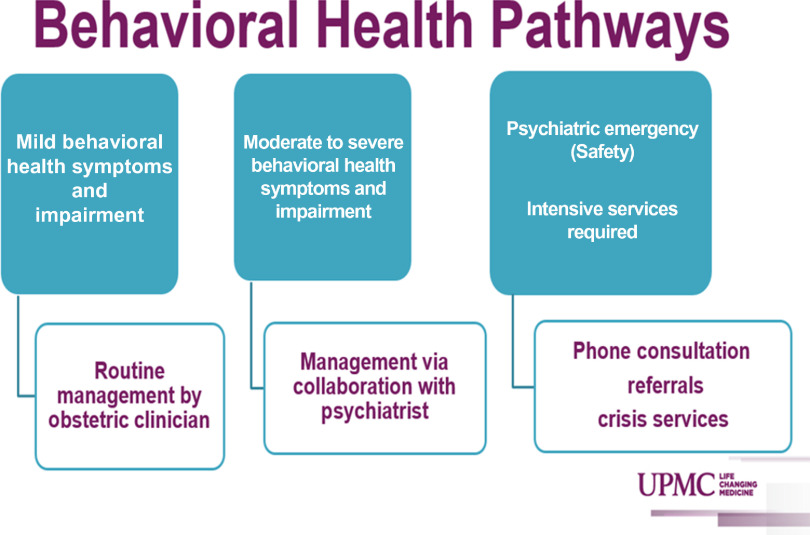
Example of spectrum of care offered via perinatal collaborative care.

The original randomized trials (Unutzer *et al*., 2002; Archer *et al*., 2012; Thota *et al*., 2012) and implementation studies (Grypma *et al*., 2006; Reiss-Brennan *et al*., 2010; Solberg *et al*., 2010; Zivin *et al*., 2010) of the CC model focused on major depressive disorder. However, studies have demonstrated benefit of the CC model with other psychiatric morbidities such as anxiety disorders (Roy-Byrne *et al*., 2010), bipolar disorder (van der Voort *et al*., 2015), and substance use disorders (Watkins *et al*., 2017). Clear delineation of the scope of care provided is essential to ensure appropriate CC staffing (Figure 1). As many obstetric providers are not trained in managing mental illness other than uncomplicated depression and anxiety, the perinatal psychiatrist will need to be able to primarily manage a subset of referred women with more complex or severe mental illness. Conversely, systematic exclusion of these women from the CC program requires clear advanced communication to the referring obstetric providers regarding the scope of care that the CC program will provide. Furthermore, it should be recognized that women may be referred to the program with perinatal depression suspected by their obstetric providers but ultimate diagnoses of bipolar disorder are made. A detailed plan should be made for management of these clinical scenarios. It should also be recognized that complicated inclusion or exclusion criteria for the CC program may serve as a barrier to obstetric provider referrals.

### Population-based tracking

Measurement-based treatment to target is one of the core principals of CC. There are various options for patient registry tools, each with unique challenges and benefits. The AIMS Center at the University of Washington offers two resources available for use (http://aims.uw.edu/resource-library/aims-caseload-tracker). The Epic EMR system has developed Epic Health Planet as a population management system that can be used within CC programs. Other web-based, HIPAA-compliant data servers, such as REDCap, with capacity to e-mail mental health screens can also be used.

### Clinical care algorithms

Clinical protocols for the approach to screening, response to positive screens, initiation of evidence-based brief psychotherapy and/or antidepressants, and dose adjustment are helpful to develop and share between the CC team and obstetric providers. Evidence-based flow sheets that succinctly illustrate key concepts can be developed in collaboration with obstetric stakeholders. While individualized care plans may be developed, standardizing the general approach to the planned stepped care algorithm will foster obstetrician comfort with screening and primary pharmacotherapy management. Examples of algorithms used in Northwestern Medicine’s CC program (COMPASS) are included in the Appendix.

### Funding

CC is a relatively new way of delivering care, and identifying available funding is beneficial to launch such innovation. A compelling needs assessment and presentation of the evidence base may persuade organizational leaders to fund a CC program. Many aspects of the CC model had been subject to limited opportunities for reimbursement, but newer adoption and expansion of CC billing codes are facilitators of implementation. The specific tasks that fall under the rubric of these time-based billing codes are described below.

Population-based care and measurement-based treatment to target require rigorous patient tracking. While freely available existing spreadsheets and tracking systems have been developed as described above, linkage of these clinical data to any existing EMR system can be costly. Furthermore, active patient reminders, surveillance for completion of screens, and communication regarding stepped care recommendations is time-consuming. In October 2017, Centers for Medicare and Medicaid Services approved Medicare reimbursement for these essential CC services (Oquendo, 2016). As more commercial payers have followed suit, reimbursement for these services may facilitate financial stability of perinatal CC programs.

Another cost is coverage of mental health care in the setting of insurance non-alignment between obstetric and mental health teams. As described previously, obstetricians are less likely to engage in CC when only a subsection of their patients are eligible for behavioral health care in the CC program, and this is particularly true in the nuanced setting of identifying mental health insurance benefits. Optimization of insurance alignment and/or funding to support empiric care provision for women whose insurances do not allow for the CC mental health care provision improves the chances of successful implementation.

One additional area of revenue generation by the care manager, when CC billing codes are unavailable, is via clinical evaluation and evidence-based brief psychotherapy provision. As described above, care manager licensure to complete these tasks is beneficial programmatically. Nevertheless, given the myriad responsibilities of the care manager in meeting CC standards, the amount of time spent delivering therapy needs to be carefully balanced with the population-level coordination needs of the program.

### Physical space

Considerations must be made both for the location of the care manager as well as for any direct psychiatric care that will be provided within the CC program clinic. Ideally, CC is co-located care, with the care manager, perinatal psychiatrist, and therapist located within the prenatal offices. Co-location of the care manager should be prioritized as it affords ongoing programmatic awareness, ‘curb-side’ consultations, and warm patient handoffs to occur. If co-location is not an option, it is critical to establish reliable communication channels between the collaborating providers, such as a direct phone number, pager, secure messaging tool, and/or regular meetings.

## Program launch

### Creating awareness

While obstetric providers will have been involved in the planning process, ensuring awareness of the program’s launch is essential. Methods of systemic notification of stakeholders include newsletter announcements, departmental/practice meetings, and/or grand rounds. Essential contact information (e.g., phone number, pager, office hours) for members of the CC team should be included in these communications ensuring that front desk employees, medical assistants, nurses, doctors, and office administrators have updated programmatic information. Professional and stigma-reducing brochures or fliers give credence to a new program and can be placed in patient exam rooms and physician workspaces as a talking point and take-home resource for patients. The clinical care algorithms developed in the planning phase should be made readily available for quick reference by obstetric providers.

### Ensuring the entire obstetric care team is aware and engaged

Launch time is the perfect opportunity to engage *all* members of the patient experience in the shared vision for CC. Front desk staff, medical assistants, and nurses are indispensable to workflow and programmatic success. For example, front desk personnel are responsible for correctly scheduling new types of patients and ensuring billing information is accurate. Their awareness and engagement in programmatic launch enables seamless registration and claims processing. Medical assistants are often involved in the initial depression screen administration, can also universally offer patient educational materials in new patient packets, and assist with warm handoffs during an in-office referral. Obstetric office nurses often respond to patient calls or messages and can be pivotal in initiating successful referrals. They are an important referral source in assisting the CC team with follow-up and engagement. Tailored messaging to all members of the team regarding the launch date and their role in the program will cultivate the needed team approach to optimizing patient care.

### Clinical care team meetings

A core tenet of CC is population-based care, engaging mental health specialists in providing caseload-focused consultation. Meeting duration can be tailored to the complexity of cases discussed but in general should occur weekly to ensure that women engaged in the CC program are being actively managed. To facilitate attendance of all CC team members at these weekly meetings, some programs conduct the meetings via video conference. Prior to each meeting, the care manager reviews the registry to identify women who are not improving according to anticipated trajectories as well as any other complicated cases. Preparation for these case review meetings ensures a systematic review of patient engagement and symptom assessment, allowing the care manager to provide targeted outreach to women who have not engaged in screenings or care.

In addition to the care manager, these clinically focused meetings should include all CC mental health providers (i.e., psychiatrist(s) and behavioral health therapist(s)) as well as, ideally, obstetrician stakeholder(s) to ensure the program’s responsiveness to the needs of the obstetric clinicians. A multidisciplinary approach allows for rich discussion and development of plans tailored to the perinatal context. Care plans developed as a result of these meetings are then communicated back to patients and their obstetric providers to enable stepped care plans to be implemented.

### Documentation

An integrated EMR is an enormous asset to CC. Ideally, behavioral health encounters are visible to all providers in the same medical record as obstetric care. Requiring obstetric providers to ‘break the glass’ or otherwise pass by a fire wall between obstetric and mental health notes decreases communication between teams and increases stigma surrounding mental health treatment. A joint record system enables obstetricians to visualize the behavioral health intake notes by the care manager. These notes include the initial discussion with each new patient, whether in-person or phone-based. This note includes basic information about the patient’s symptoms, mental health history, treatment preferences, treatment plan, and intake screening scores. This is a quick reference point for all members of the care team.

Other useful components of the EMR include identification of a centralized location for depression screens, such as a flow sheet, that enables both mental health and obstetric providers to visualize symptom trajectories over time. In addition, utilization of a centralized problem list, briefly outlining the diagnosis and treatment plan, allows for quick reference to the shared care plan with each patient encounter.

### Referrals for care outside of the CC program

As the scope of each program is refined, some women may be better served with a different level of care (e.g., intensive outpatient care) or may require ongoing care outside of the perinatal time frame. Additionally, women may prefer to receive ongoing care during times or in locations that cannot be accommodated within the CC program. Identification of trusted referrals can facilitate the most appropriate linkage. While some programs do not continue to track in their registry women primarily receiving mental health care elsewhere, others do track such women to serve as a safety net by continuing to engage in systematic symptom screening with potential re-engagement in CC outpatient treatment at later dates.

## Program growth and sustainment

### Administrative meetings

Outside of the weekly clinical care meetings, regular meetings with the administrative teams in the beginning are essential, as many logistical issues will be identified in the early launch period. These meetings may become less frequent as the program becomes more established. Planned meetings with other CC programs can allow programs to compare strengths and growth areas, learn about new resources inside and outside the medical system, discuss potential improvements, and nurture new programs through guidance.

### Treatment to target

**Figure 2.**
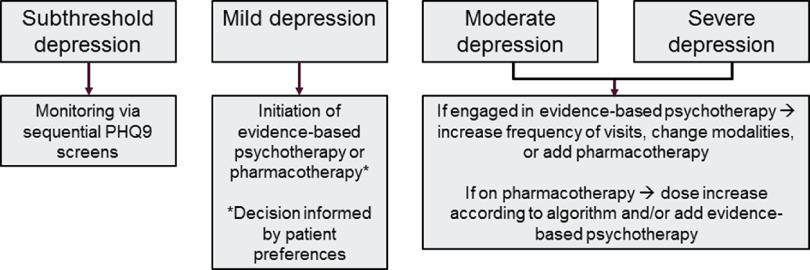
Stepped care model.

A central tenet of CC is measurement-based treatment to target: ensuring that each patient’s treatment plan articulates the clinical goals and that evidence-based surveillance tools inform treatment changes. The elements central to this goal have been previously described, particularly screening protocols for patients engaged in treatment, documenting in the medical record and a secure patient tracking tool, and regular communication with both patients and providers. This overarching principal is also called ‘stepped care,’ and a general algorithm to achieve this goal is depicted in Figure 2.

**Figure 3.**
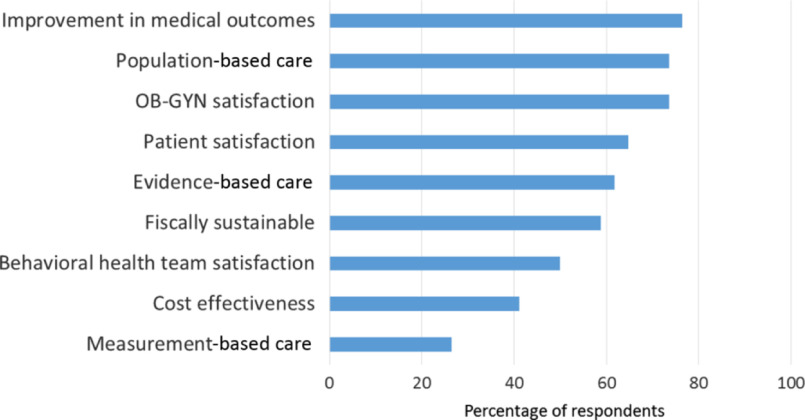
Core collaborative care principles reported by survey respondents.

### Patient engagement

Despite the team’s best efforts, some patients will be lost to follow-up. The larger the patient volume, the more important it is to have protocols in place for handling poor patient engagement. In Northwestern’s COMPASS program, about 25% of referred patients never enroll, generally due to not responding to repeated outreach attempts or due to denying need for mental health services upon discussion with the care manager. A clear communication plan is essential to ensure the obstetric team is aware that enrollment did not occur. The obstetric team can continue with supportive psychoeducation to facilitate linkage as well as curbside consultation to the obstetric team.

Even once women are enrolled, some women engage less with the treatment plan as they clinically improve or as time elapses. This can be prominent postpartum given the newly competing priorities as women introduce the demands of newborn/child care (Bowen *et al*., 2012). A defined protocol to address this challenge will optimize communication between care teams. To assist with engagement challenges, a care coordinator may benefit from basic training in Motivational Interviewing (Rubak *et al*., 2005; Grote *et al*., 2014). Ultimately, the expectation with postpartum women is achievement of clinical stability to enable transition to maintenance treatment via a primary care physician or a community mental health program. Ensuring a seamless transition is essential in maintaining patient engagement in her mental health care maintenance.

### Continued communication with stakeholders

Continued communicating with stakeholders is essential for gaining feedback for healthy growth and development of a perinatal CC program. Some examples of specific communication pathways are described below.

1. *Obstetric providers* – Ongoing attendance at clinical practice meetings to give program updates and continuing education fosters continued engagement in CC. Some providers (especially those demonstrating confusion or resistance to the CC approach) may benefit from individual follow-up with program leaders. As the program grows, it could also be helpful to share a ‘report card’ with obstetric clinics to demonstrate the positive impact of the CC model. This could include number of referrals, conversion to enrolled patients, prenatal depression screening rates, and treatment outcomes of their patients.
2. *Administration* – Meetings between administrators from both the psychiatry and obstetric practices are useful to address proactively changes in clinical or financial work flows.
3. *Funders* – New CC programs are likely to have some kind of financial support from a grantor, foundation, or organizational leadership. Collecting data and success stories throughout implementation enables the program to express appreciation and may open opportunities for ongoing support.
4. *Patients* – ‘Patient-centered team care’ is a core principal of CC. Developing opportunities for formal and informal patient feedback will ensure the program is meeting the clinical needs of referred women.

### Financial sustainment

Development of a comprehensive financial sustainability plan should occur before launching a CC program. Assessment of benchmarks, via regular check-ins with administrators, to reassess progress toward financial goals, is essential to ensure mutual agreement on trajectories. For some programs, this will mean simple budget neutrality. Some programs may even be expected to reap a profit for the health center sponsoring the program. And in other health centers, leaders may recognize that the worth of CC goes beyond profitability. The expectations of administrative supporters will likely dictate productivity needs of the program and ongoing conversations will ensure clarity about these expectations.

### Training the next generation of providers

Core to the COMPASS program is an investment in the education of the next generation of providers. To that end, the CC psychiatrist role is staffed by the women’s mental health fellow(s), supervised by psychiatry faculty. Not only does this enhance their education through rich exposures to various clinical scenarios, but it fosters an appreciation for health systems programming that may expand opportunities for CC implementation within their ultimate faculty positions. Residents in obstetrics and gynecology are a core referral group. Their involvement facilitates teaching obstetricians-in-training about effective perinatal mental health treatment, providing them with the requisite skills to deliver evidence-based mental health care to their patients moving forward. Finally, students interested in future careers in medicine, psychology, or social work can be helpful with population management, chart abstractions, and data entry while gaining exposure to CC systems.

## Current landscape of perinatal behavioral health models

To understand the current landscape of provision of behavioral health in obstetrics and gynecology outpatient settings, a 16-question survey (via Qualtrics anonymous survey link) was disseminated to over 100 perinatal mental health practitioners through The International Marcé Society for Perinatal Mental Health and the Postpartum Support International Reproductive Psychiatry listservs between June and October 2017. The survey was entitled ‘Integrated Behavioral Health in OB-Gyn Outpatient Settings’ and was designed to assess broadly practitioners engaged in program development. There were only 34 survey respondents, suggesting that the focused integration of behavioral health is uncommon in perinatal settings. The majority of programs (73.5%) were less than five years in the making; of those, 52% were new in the past two years. Twenty-seven respondents provided their clinic location and professional specialty. Twenty-three of twenty-seven (85.2%) were from the United States; the other four respondents (14.8%) were from Canada, Brazil, The Netherlands, and India. Psychiatrists were most highly represented among respondents (*n* = 15; 55.6%), followed by behavioral health therapists and psychologists (*n* = 6; 22.2%), obstetric and gynecologic physicians (*n* = 2; 7.4%), nurse practitioners (*n* = 2; 7.4%), a pediatrician-psychiatrist (*n* = 1; 3.7%), and an maternal fetal medicine physician (*n* = 1; 3.7%).

*Meetings:* Two-thirds of programs (67.6%) held 10 or fewer planning meetings with stakeholders and a little over half (57.6%) planned for six months or fewer. Fifty percent of practices maintained clinical meetings at a frequency of 1 to 4 times per month, with the other half meeting much less frequently, and 17.6% not meeting at all. These data suggest that CC is not a common model of care.

*Clinic staffing:* The vast majority of our respondents’ clinics (91%) were staffed by a psychiatrist – indicating that most perinatal clinics are practicing a modified integrated care, rather than the CC model described in primary care. With the care manager’s critical role in CC, it is interesting to note that only 38% of respondents had at a care manager in their clinic, even though the majority of non-billable hours were spent in care management and referral consultation. Given that therapists often fulfill both the care manager and therapist role, it is reassuring to note that 73.5% of respondents reported having at least one therapist. 35.3% of clinics had a behavioral health nurse. The fact that staffing in clinics is so different from the evidence-based CC model may indicate that clinics are operating with meager resources, that obstetric providers are reluctant to engage in full CC, or that other unconsidered factors may be involved.

*Prioritization of clinic principles:* Respondents prioritized (>70%) obstetrician and gynecologist satisfaction, population-based care, and improvement in medical outcomes (Figure 3). Measurement-based care was the lowest priority (26.5%). Approximately, 40% of respondents noted ‘Consistent’ or ‘To some extent’ use of systematic case review and stepped care treatment adjustment for non-responsive patients. Nearly, 15% of respondents used neither.

*Instruments:* EPDS, PHQ-9, and GAD-7 were the most commonly used scales utilized in perinatal CC programs.

*Clinic characterization by population served:* Over 50% of programs served only perinatal women (defined as pregnancy out to six to twelve months postpartum, or a flexible end period based upon needs), whereas 20% and 18% of programs served either obstetric or gynecologic patients or women with reproductive-related behavioral health disorders, respectively.

*Referrals and triage:* Sources of patient referral were highly variable, ranging from (most to least common) one obstetric practice in one location, several practices in multiple locations, anyone who calls to schedule an appointment, and one obstetric practice with multiple locations. There were diverse methods for triaging referrals with phone triage, no triage, and therapist first assessment (i.e., warm handoffs).

*Treatment duration*: Short-term treatment seemed to be the norm, with approximately 40% of patients having only 1–3 visits, and 60% having four or more visits for psychotherapy and/or medication management.

*Fiscal:* Nearly 60% of programs collected health insurance fees for behavioral health services, but many of these also relied upon obstetric (42%), psychiatry (36%) department, federal (12%), and non-federal (36%) grant funding.

*Visit frequency:* 30–50% of patients were seen by behavioral health in a total of 1–3 visits, suggesting that many women coming to these clinics have short-term needs or do not engage well in treatment.

In summary, based on these survey results integrated behavioral health programs within obstetrics offices are new, supported by medical departments, and vary in the frequency of stakeholder meetings to prepare for their clinical launch. Few programs seem to leverage the CC model, based on a reported reduced emphasis on measurement-based care, systematic case review, stepped care protocols, and weekly case review meetings. Culture shift occurs slowly with facilitators that include obstetrician champions, financial incentives through insurance companies and departmental support, perseverance, and compiling shared experience to improve individual efforts in this realm.

## Conclusions

Significant momentum exists in implementing integrated behavioral health in the perinatal setting, but programs often fall short of true evidence-based CC and thus their anticipated effectiveness may diverge from what would be anticipated from a program with higher fidelity to the CC model. The tenets outlined here will serve as a resource for clinicians interested in starting CC programs or transitioning their existing program to utilization of a CC model. As our survey indicates, health care providers can and will start integrated care programs even when funding is limited and not all the elements listed above can be achieved; sometimes a small pilot program can help both to improve stakeholder engagement and to collect outcomes data that can be used to secure additional funding. While perinatal CC has been proven efficacious, ongoing research will identify whether the implementation strategies described herein translate to effectiveness – and whether the modified versions of these strategies currently in play at existing programs can also be effective.

## Supplementary material

For supplementary material accompanying this paper visit http://dx.doi.org/10.1017/S1463423620000110. click here to view supplementary material
