# Supplementary figures and images for: Implementation of perinatal collaborative care: a health services approach to perinatal depression care
Source: Prim Health Care Res Dev. 2020 Sep 10;21:e30. doi: 10.1017/S1463423620000110 (PMC7503171; doi:10.1017/S1463423620000110)

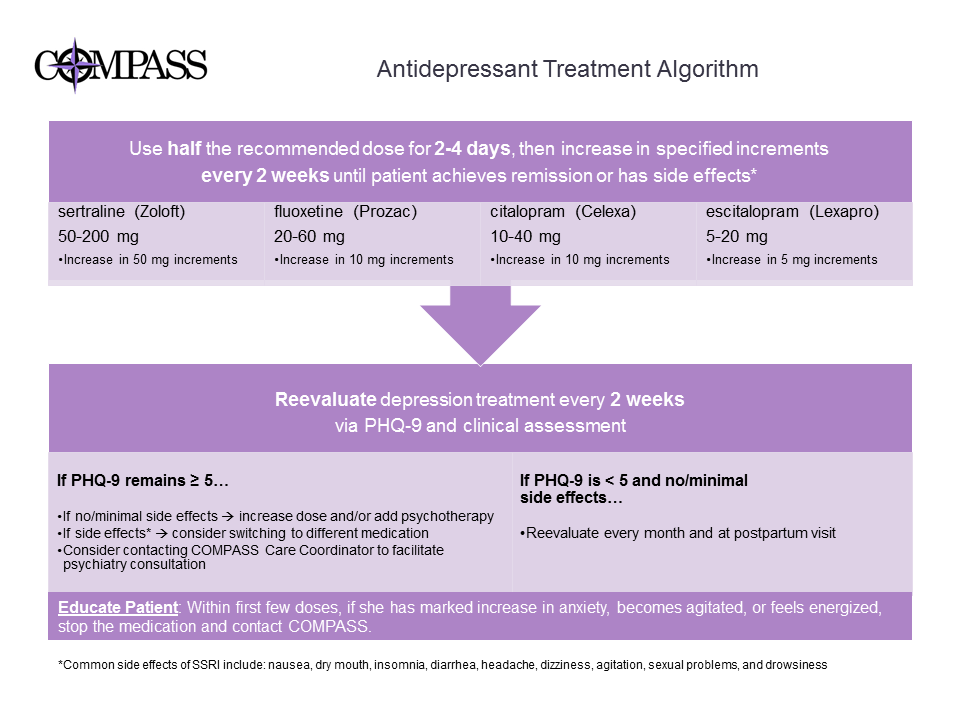

Supplement: Supplementary file 1 [file S1463423620000110sup.zip › S1463423620000110sup002.tif]

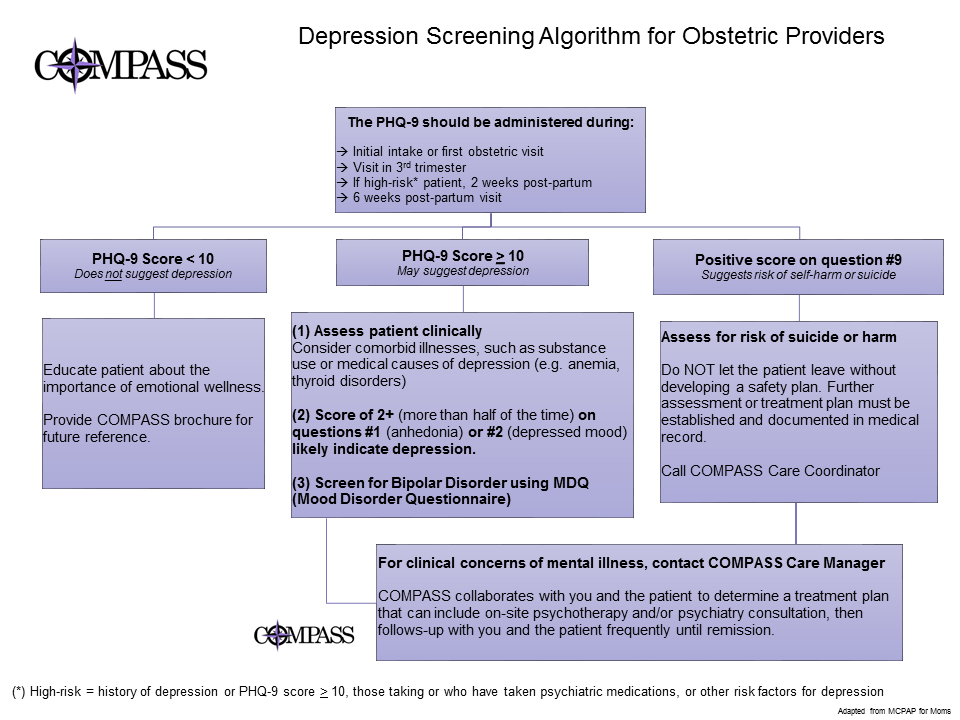

Supplement: Supplementary file 1 [file S1463423620000110sup.zip › S1463423620000110sup003.tif]
